# Insecticide resistance mutations of *Anopheles* species in the Republic of Korea

**DOI:** 10.1371/journal.pntd.0012748

**Published:** 2025-01-07

**Authors:** Jiseung Jeon, Heung Chul Kim, Terry A. Klein, Hojong Jun, Kwang Shik Choi

**Affiliations:** 1 Department of Biology, College of Natural Sciences, Kyungpook National University, Daegu, Republic of Korea; 2 School of Life Sciences, BK21 FOUR KNU Creative BioResearch Group, Kyungpook National University, Daegu, Republic of Korea; 3 U Inc., Daesakwan-ro 34-gil, Yongsan-gu, Seoul, Republic of Korea; 4 Force Health Protection and Preventive Medicine, Medical Department Activity-Korea/65th Medical Brigade, Unit 15281, Pyeongtaek, Republic of Korea; 5 Department of Medical Environmental Biology and Tropical Medicine, School of Medicine, Kangwon National University, Chuncheon, Republic of Korea; CNRS: Centre National de la Recherche Scientifique, FRANCE

## Abstract

The number of reported malaria cases transmitted by *Anopheles* mosquitoes in the Republic of Korea (ROK) increased from 420 in 2022 to 746 in 2023, a 77.6% increase. Eight *Anopheles* species are currently reported in the ROK, including six species belonging to the *Anopheles* Hyrcanus Group and one species each belonging to the Barbirostris Group and Lindesayi Group. However, studies on insecticide resistance in the ROK has predominantly concentrated on *Anopheles sinensis* or more broadly, members of the Hyrcanus Group. Reported differences in vector competence and ecological characteristics of mosquito species in the ROK highlight the importance for conducting accurate evaluations of insecticide resistance for each of the *Anopheles* species for informing the potential efficacy of vector control to reduce malaria transmission. All eight species of *Anopheles* mosquitoes were collected in/near the demilitarized zone (DMZ), a malaria high-risk region in the ROK. Additional specimens were collected in Seoul [Yongsan US Army Garrison (USAG)] and Pyeongtaek (Humphreys USAG) where malaria risks are much lower. *Anopheles* mosquitoes were identified to species using a multiplex PCR method and then evaluated for the presence of acetylcholinesterase-1 (*ace-1*) and voltage-gated sodium channel (*vgsc*) regions to identify mutations linked to insecticide resistance. Analysis of the *ace-1* region identified insecticide resistance alleles in four species of the Hyrcanus Group (*An*. *sinensis*, *An*. *kleini*, *An*. *belenrae*, and *An*. *pullus*), while *ace-1* resistance alleles were not observed in the other four species. The screening of the *vgsc* gene fragment confirmed the presence of resistant alleles only in *An*. *sinensis* (considered a poor malaria vector) and *An*. *kleini* (a primary malaria vector) in the ROK. This study represents a preliminary investigation of insecticide resistance mutations across all *Anopheles* species in the ROK. These findings are crucial in advancing mosquito control strategies to mitigate future malaria infections.

## Author summary

Despite national efforts to suppress malaria outbreaks, there has been a recent increase in the number of vivax malaria cases in the Republic of Korea (ROK). Insecticides are the most cost-effective and efficient approach for vector control. However, mosquito populations consistently exposed to these insecticides experience significant selection pressures to develop resistance. While insecticide resistance plays a crucial role in effective vector control, current research on insecticide resistance in the ROK has primarily concentrated on *Anopheles sinensis* that is considered to be a poor malaria vector. This study examined the presence of insecticide resistance-related mutations in all *Anopheles* mosquito species in the ROK, aiming to enhance the future development of effective malaria vector control strategies. Screening for the G119S mutation in the acetylcholinesterase-1 gene, associated with organophosphate insecticides, and the identification of knockdown resistance mutations in the voltage-gated sodium channel gene demonstrated the presence of previously unrecognized resistance mutations in *Anopheles* species in the ROK. We also observed variations in seasonal insecticide resistance among each of the species. These findings indicate that the rotation and/or combination of insecticides are necessary for effective vector control, thereby providing a valuable strategy for future malaria control efforts.

## Introduction

*Plasmodium vivax* is the only endemic human malaria transmitted by *Anopheles* spp. in the Republic of Korea (ROK) [1, 2]. The World Health Organization (WHO) designated the ROK as a key country for malaria elimination [3,4], and the ROK is implementing diverse initiatives to attain zero malaria cases by 2028 [2]. Despite national efforts to control malaria infections, the number of cases increased from 200–300 cases during 2020–2022 to 746 in 2023. Nearly all vivax malaria cases were reported adjacent to or as a result of exposure near the demilitarized zone (DMZ) in northern Gyeonggi and Gangwon provinces [5].

There are six species belonging to the Hyrcanus Group (*An*. *sinensis* sensu stricto Wiedemann; *An*. *kleini* Rueda; *An*. *belenrae* Rueda; *An*. *pullus* Yamada; *An*. *sineroides* Yamada; and *An*. *lesteri* Baisas and Hu), and one each belonging to the Barbirostris Group (*An*. *koreicus* Yamada and Watanabe) and Lindesayi Group (*An*. *lindesayi* Giles) present in the ROK [6,7]. Studies demonstrated various degrees of malaria vector competence [8,9,10,11]. Based on recent studies, *An*. *lesteri* and *An*. *kleini* are regarded as primary malaria vectors, while *An*. *sinensis*, *An*. *belenrae*, and *An*. *pullus* are poor vectors [8,9,10,11]. Continuous surveillance and studies on the seasonal occurrence patterns and ecological features of each species (such as habitat and preferred hosts), is essential for developing effective malaria control programs.

The most cost-effective method for managing mosquitoes involves the application of pesticides, typically organophosphates and pyrethroids. However, mosquito populations subjected to persistent insecticide exposure face selective pressure to acquire insecticide-resistant traits. This phenomenon progressively emerges as a significant challenge that hinders the implementation of effective control measures that impact both vectors of disease-causing pathogens and agricultural pests [12,13]. The mechanisms that contribute to insecticide resistance include target site mutations, metabolic detoxification, reduced penetration, and behavioral resistance [13]. Among these mechanisms, target site mutations result from alterations in the codons of specific genes. A gene recognized for its correlation with resistance to organophosphate and carbamate insecticides is acetylcholinesterase-1 (*ace-1*). A specific mutation (119S) occurring in the 119th codon that results from GGC (glycine) changing to AGC (serine) has been identified as a cause of insecticide resistance in *Culex pipiens* and *An*. *gambiae* [14]. Resistance to pyrethroid insecticides is linked to the voltage-gated sodium channel (*vgsc*) gene and is attributed to a specific point mutation at the 1014th codon (1014L) in *Cx*. *pipiens*, *An*. *gambiae* and *An*. *coluzzi* [15,16]. Several knockdown resistance (*kdr*) mutations have been documented in pyrethroid insecticides. In *An*. *gambiae*, point mutations at the 1014F and 1014S codons have been identified, where the 1014th codon, originally encoding leucine, is changed to phenylalanine and serine, respectively, a situation that can potentially lead to significant public health challenges [17,18,19,20,21,22]. The 119G (*ace-1*)/1014L (*kdr*) nomenclature does not accurately represent the codon numbers in *Anopheles*; for instance, *An*. *gambiae* corresponds to codons 280G (*ace-1*) and 995L (*kdr*) [23]. However, since eight species were included in this study and determining the correct codon numbers is challenging, conventional 119G (*ace-1*)/1014L (*kdr*) nomenclature is used.

The 119S mutation in *ace-1* and *kdr* mutations in the *vgsc* gene fragments in *Anopheles* spp. has been reported in the ROK [24,25,26,27,28,29,30]. Studies in the ROK primarily concentrated on investigating either *An*. *sinensis* or broadly for members of the Hyrcanus Group to identify resistance mutations [24,25,27,28,29]. These studies validated the presence of *ace-1* (119S) and *kdr* (1014F, 1014C—leucine to cysteine) mutations in *An*. *sinensis*. Limited sample sizes demonstrated the presence of the *kdr* mutations (1014F, 1014C) exclusively in *An*. *sinensis*, with no mutations detected in the remaining five species within the Hyrcanus Group [26]. Later the *kdr* mutation (1014F) was shown to be present in *An*. *belenrae* [30]. Nevertheless, insufficient numbers of *Anopheles* spp., other than *An*. *sinensis*, does not address the issue of pesticide resistant alleles in competent malaria vectors in the ROK [30].

Members of the Hyrcanus Group in the ROK poses morphological challenges. However, specific identification can be accomplished using a multiplex polymerase chain reaction (PCR) method based on the internal transcribed spacer2 (ITS2) region [31,32]. Similarly, conventional sequencing methods, such as Sanger sequencing that is widely regarded as the gold standard for detecting target site mutations at the individual level, are both labor intensive and costly [25,28,33,34,35]. The precise identification of insecticide resistance mutations at the species/individual level is crucial for developing comprehensive and effective vector control measures in the ROK, particularly as malaria cases continue to rise. This study analyzed mutations associated with insecticide resistance at the species/individual level within the DMZ, a significant area of malaria risk, and in two localities south of the DMZ. The aim was to ascertain the prevalence of resistance mutations in each of the eight *Anopheles* species found in the ROK.

## Methods

### *Anopheles* collection

**Fig 1:**
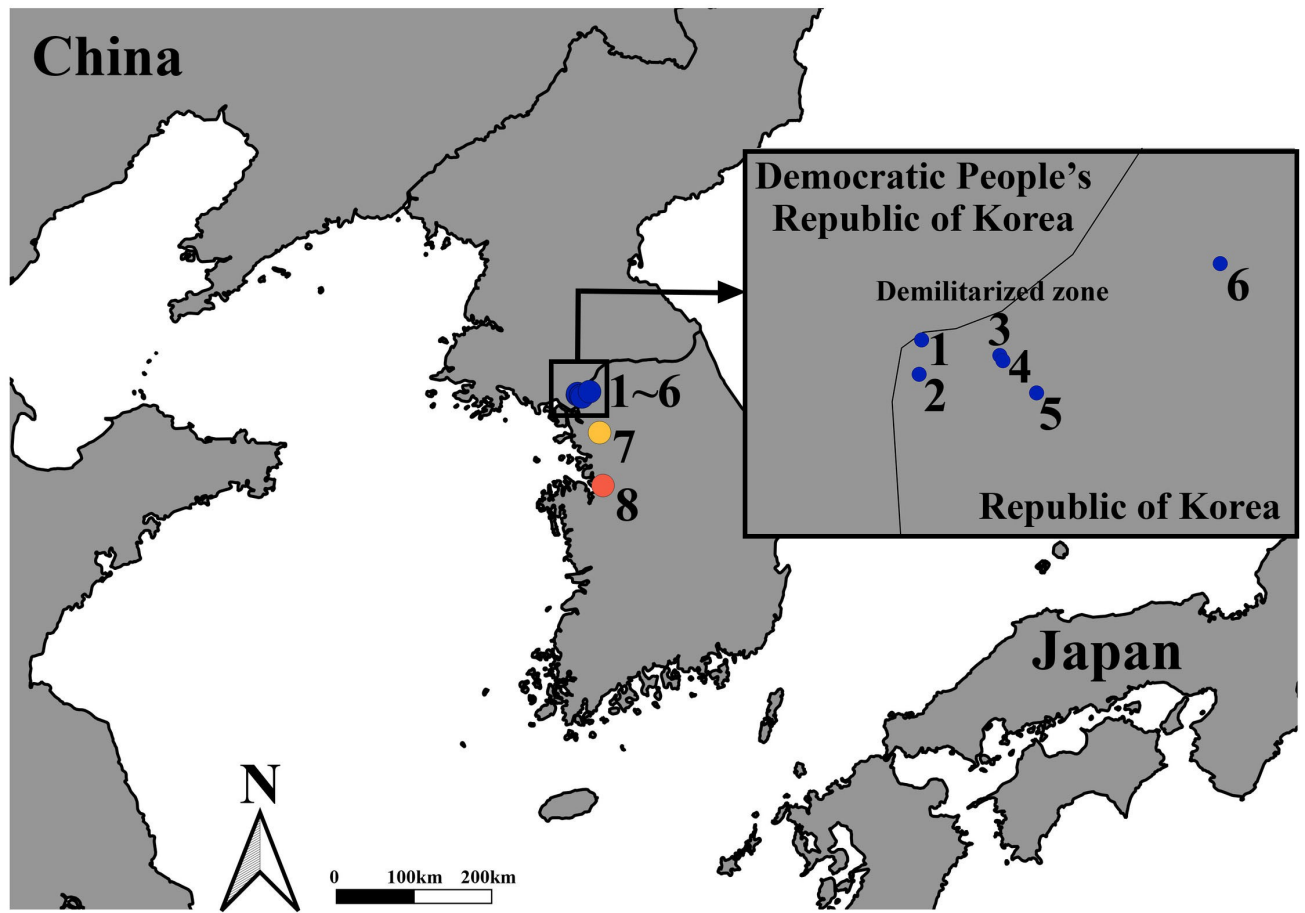
Mosquito collection sites from April through October 2021. 1. Neutral Nations Supervisory Commission camp (<10 m from the DMZ), 2. Daeseong-dong (village inside the DMZ), 3. South gate (entrance to the DMZ), 4. Camp Bonifas, 5. Warrior Base training area, 6. Dagmar North training area, 7. Seoul (Yongsan US Army Garrison), and 8. Pyeongtaek (Humphreys US Army Garrison). This map was created using QGIS version 3.26.3 (https://www.qgis.org/ko/site) and Natural Earth (https://www.naturalearthdata.com/downloads/10m-cultural-vectors; http://www.naturalearthdata.com/about/terms-of-use).

Mosquitoes were collected from April through October 2021 in/near the DMZ, a malaria high-risk region in the ROK that borders the Democratic People’s Republic of Korea (North Korea). Collection areas included: (1) Neutral Nations Supervisory Commission (NNSC) camp, 37°57′17.19″ N; 126°40′47.91″ E, (2) Daeseong-dong (village inside the DMZ), 37°56′28.31″ N; 126°40′37.38″ E, (3) South gate entrance to the DMZ, 37°56′03.53″ N; 126°43′15.46″ E, (4) Camp Bonifas, 37°55′55.25″ N; 126°43′21.73″ E, (5) Warrior Base training area (TA), 37°55′03.96″ N; 126°44′29.74″ E, and (6) Dagmar North TA, (37°58′29.85″ N; 126°50′40.88″ E). Two additional collection sites south of the DMZ included Seoul [Yongsan US Army Garrison (USAG), 37°31′56.2″ N; 126°58′53.4″ E] and Pyeongtaek [Humphreys USAG, 36°57′19.9″ N; 127°01′41.4″ E] (**Fig 1**). Mosquitoes were collected using Mosquito Magnets (Woodstream Corp., Lancaster, PA, USA) as previously described [36]. Mosquitoes were collected biweekly and transported to the Entomology Section, Force Health Protection and Preventive Medicine, 65^th^ Medical Brigade, located at Humphreys USAG. Mosquitoes were stored at -80°C, subsequently identified to species (*An*. *sineroides*, *An*. *koreicus*, and *An*. *lindesayi*) or *Anopheles* Hyrcanus Group, and then returned to the ultra-low temperature freezer. Selected mosquito specimens were transported on dry ice to Kyungpook National University (Daegu, ROK) where they were stored at -70°C until used.

### Species identification

*Anopheles* spp. were identified to species using a multiplex PCR method that identified all eight species using the Clear-S Quick DNA Extraction kit (InVirusTech, Gwangju, ROK), following the manufacturer’s protocol [32,36]. In short, primers used to identify all eight species included: (universal forward primer (5′-ATC GAT GAA GAC CGC AGC TA-3′), species-specific reverse primers (*An*. *sinensis* (1112 bp): 5′-TAG GGT CAA GGC ATA CAG AAG G-3′; *An*. *koreicus* (925 bp): 5′-TAT CGT GGC CCT CGA CAG-3′; *An*. *lindesayi* (650 bp): 5′-ACC ATC TAC TGC CTG AAC GTG-3′; *An*. *kleini* (527 bp): 5′-TTT GTT GAT AAC TTG TAT CGT CCA TC-3′; *An*. *lesteri* (436 bp): 5′-CAG TCT CTT GCA GCC CAT TC-3′; *An*. *sineroides* (315 bp): 5′-CGC GCA CGC TCA GAT ATT-3′; *An*. *belenrae* (260 bp): 5′-TGT CCT AGG CGG TTA TCA ACA-3′; and *An*. *pullus* (157 bp): 5′-CGG CGT AGT TTA TTG TGT ATA ACA TC-3′)) [32]. The reaction mixture for PCR amplification (total volume: 12.5 μL) included: 1× PCR buffer, 0.2 mM dNTPs, 0.4 μM of each primer, 0.5 units *Taq* hot-start DNA polymerase (TaKaRa, Shiga, Japan), and 0.5 μL extracted genomic DNA. PCR cycling conditions were as follows: initial denaturation at 94°C for 5 min followed by 35 cycles at 94°C for 30 sec, 55°C for 30 sec, 72°C for 2 min, and final extension at 72°C for 5 min. The PCR products were separated using a 1.5% agarose gel.

### DNA sequencing

After specific identification, DNA sequencing was conducted to identify pesticide resistant alleles using PCR methods and Sanger sequencing to identify 119S (*ace-1*) and *kdr* mutations in the *vgsc* gene. Primers previously employed by Qin *et al*. [37] to identify the 119S mutation in the *ace-1* gene of *An*. *sinensis* were utilized with minor modifications: (forward primer: 5’-GAC CAT GTG GAA CCC GAA C-3’; reverse primer: 5’-ACC ACG ATC ACG TTC TCC TC-3’). The reaction mixture for PCR amplification (total volume: 25 μL) included 1× PCR buffer, 0.2 mM dNTPs, 0.4 μM of each primer, 0.5 units *Taq* hot-start DNA polymerase (TaKaRa, Shiga, Japan), and 1 μL extracted genomic DNA. The PCR cycling conditions were as follows: initial denaturation at 94°C for 5 min followed by 35 cycles at 94°C for 30 sec, 54°C for 30 sec, 72°C for 30 sec, and final extension at 72°C for 5 min.

Primers previously described to detect *kdr* mutations included: 5′ASIIS56: 5’-CGG ACT TCA TGC ACT CCT TCA-3’; 3′ASIIS56: 5’-TTA GCG CAT TTG CTA CGT TC-3 [25]. The reaction mixture for PCR amplification (total volume: 25 μL) included 1× PCR buffer, 0.2 mM dNTPs, 0.4 μM of each primer, 0.5 units *Taq* hot-start DNA polymerase (TaKaRa, Shiga, Japan), and 1 μL extracted genomic DNA. The PCR cycling conditions were as follows: initial denaturation at 94°C for 5 min followed by 35 cycles at 94°C for 30 sec, 54°C for 30 sec, 72°C for 30 sec, and final extension at 72°C for 5 min. For *An*. *koreicus* and *An*. *lindesayi*, which are not included in the Hyrcanus Group, PCR amplification using the primers from Kim *et al*. [25] was unsuccessful. Therefore, new primers were specifically designed based on the reference sequences (GenBank accession numbers: *An*. *sinensis* (DQ334052, ON051523); *An*. *gambiae* (DQ022108, EU078897)); (LK_kdr_F: 5’-GAC TTC ATG CAT TCC TTC AT-3’; LK_kdr_R: 5’-CCG AAA TTG GAC AAA AGC A-3’). The reaction mixture for PCR amplification (total volume: 25 μL) included 1× PCR buffer, 0.2 mM dNTPs, 0.4 μM of each primer, 0.5 units *Taq* hot-start DNA polymerase (TaKaRa, Shiga, Japan), and 1 μL extracted genomic DNA. The PCR cycling conditions were as follows: initial denaturation at 94°C for 5 min followed by 35 cycles at 94°C for 30 sec, 52°C for 30 sec, 72°C for 30 sec, and final extension at 72°C for 5 min.

PCR amplification was validated using a 1.5% agarose gel, approximate amplicon sizes (*ace-1*: 190 bp; *kdr*: 350 bp); PCR products that were successfully amplified underwent direct Sanger sequencing (Macrogen, Daejeon, ROK). Following sequencing, the genomic products were aligned utilizing BioEdit version 7.2 and assessed for 119S (*ace-1*) and the *kdr* mutations in the *vgsc* gene [38]. Sequencing data obtained in this study were deposited in GenBank (GenBank accession numbers: *ace-1*: PP548002~PP548013; *kdr*: PP547989~PP548001).

### Statistical analysis

Duplication of genes associated with insecticide resistance are known in various mosquito species, and a possible duplication of the *ace-1* gene in *An*. *sinensis* has been suggested [39]. Although Hardy-Weinberg equilibrium (HWE) is not a general test for gene duplication, the HWE test can be a useful indicator for detecting gene duplications in natural populations if the duplications are at a reasonably high frequency [40]. To detect the potential for gene duplication in *Anopheles* mosquitoes, the heterozygote excess of each insecticide resistance gene was examined. The Genepop version 4.7.5 was utilized to conduct the HWE test (probability test) [41,42].

Statistical comparisons of allele frequencies for each species were conducted utilizing the Mann-Whitney U-test in the R version 4.3.3 software environment (https://www.R-project.org/).

## Results

### Species composition

A total of 489 mosquitoes belonging to eight species were identified from specimens collected from six collection sites in/near the DMZ and two collection sites south of the DMZ (**Tables 1 and S1**). Only three species (*An*. *sinensis*, *An*. *pullus*, and *An*. *lesteri*) were collected from Humphreys USAG, adjacent to Anjeong-ri (village), Pyeongtaek-si (city) and approximately 100 km from the DMZ, while only two species (*An*. *sinensis* and *An*. *lindesayi*) were collected at Yongsan USAG, located in the urban center of Seoul and approximately 60 km from the DMZ. *Anopheles kleini* and *An*. *sinensis* were the most commonly collected *Anopheles* spp. in/near the DMZ.

**Table 1:** The number of *Anopheles* spp. collected at each of the collection sites from April through October 2021.

Species	In/near the DMZ**						DMZ Subtotal	Non-DMZ***		Non-DMZ Subtotal	Grand Total
	NNSC	Daeseong-Dong	South Gate	Camp Bonifas	Warrior Base	Dagmar North		Yongsan USAG	Humphreys USAG		
*An*. *sinensis*	4	26	2	8	27	4	71	17	34	51	122
*An*. kleini****	37	25	20	14	22	7	125	-	-	-	125
Hybrid*	-	5	-	-	-	-	5	-	-	-	5
*An*. *belenrae*	11	22	4	2	7	2	48	-	-	-	48
*An*. *pullus*	13	12	7	6	5	11	54	-	22	22	76
*An*. lesteri****	-	1	1	-	1	-	3	-	14	14	17
*An*. *sineroides*	19	4	6	6	7	19	61	-	-	-	61
*An*. *koreicus*	22	2	-	1	1	-	26	-	-	-	26
*An*. *lindesayi*	2	-	4	-	-	2	8	1	-	1	9
Total	108	97	44	37	70	45	401	18	70	88	489

* Hybrid refers to *An*. *sinensis-An*. *kleini* specimens based on 527 bp and 1112 bp bands.

** NNSC = Neutral Nations Supervisory Commission Camp; Daeseong-dong = village inside the DMZ; South Gate = southern entrance into the 2 km wide southern portion of the DMZ; Camp Bonifas = ROK/US military camp adjacent to the South Gate Entrance to the DMZ; Warrior Base = US cantonment area south of the Imjin River and approximately 2 km from the DMZ; Dagmar North training area is an unimproved US military training area approximately 5 km from the DMZ.

*** Yongsan USAG is an US military installation in the urban center of Seoul and approximately 60 km from the DMZ; Humphreys USAG is adjacent to Anjeong-ri (village) in Pyeongtaek-si (city) and approximately 100 km from the DMZ.

**** The primary malaria vectors in the ROK.

**Fig 2:**
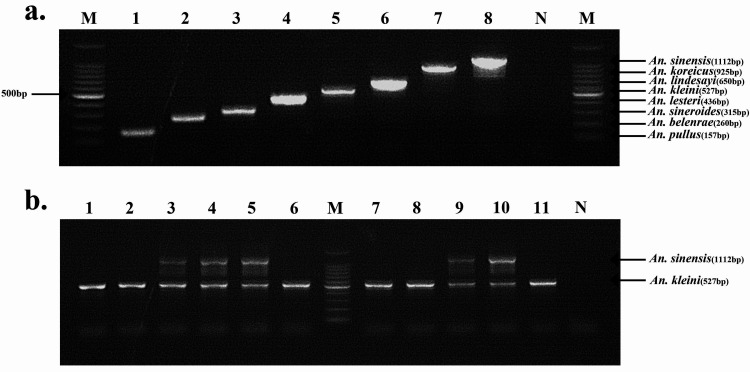
**(A)** Example of the multiplex PCR results used to identify the eight *Anopheles* species in the ROK. **(B)** Hybrids (lanes: 3,4,5,9,10) of *An*. *sinensis* (1112 bp) and *An*. *kleini* (527 bp). Lane M: 100 bp molecular ladder marker; lane N: negative control.

Double bands representing hybrids of *An*. *sinensis* (1112 bp) and *An*. *kleini* (527 bp) were observed in five individuals collected at Daeseong-dong, a village bordering the DMZ (**Fig 2**). Natural hybrids between *An*. *sinensis* and *An*. *kleini* with mixed ITS2 sequences have been previously observed in wild-caught specimens in the ROK [43]. Subsequently, laboratory studies demonstrated that *An*. *sinensis* and *An*. *kleini* hybrids produced viable offspring [44]. Each of the bands (1112 bp and 527 bp) was sequenced via gel extraction for the five *An*. *sinensis*–*An*. *kleini* hybrids (Macrogen, Daejeon, ROK). the 527 bp band and 1112 bp band aligned with *An*. *kleini* and *An*. *sinensis* sequences, respectively, after performing an NCBI BLAST analysis (GenBank accession numbers: *An*. *sinensis*: PP536553—PP536557; *An*. *kleini*: PP544155—PP544159).

### Detection of the 119S mutation in *ace-1*

Individuals of each of *Anopheles* spp. collected at the six collection sites in/near the DMZ (< 15 km) were analyzed as a single population, while individuals from Yongsan USAG and Humphreys USAG were analyzed as separate populations.

The *ace-1* gene for each of the eight species was examined to detect the presence of the 119S resistant allele (**S1 Fig**). The 119S resistant allele was identified in only four species (*An*. *sinensis*, *An*. *kleini*, *An*. *belenrae*, *An*. *pullus*) (**Table 2**). The allele frequencies of the 119S allele observed in *An*. *sinensis*, the only species that was collected at all three primary collection sites, were 0.67 (in/near the DMZ), 0.62 (Yongsan USAG), and 0.57 (Humphreys USAG). The allele frequencies of the 119S allele in the three regions of *An*. *sinensis* were not statistically significantly different (Mann-Whitney U-tests, p > 0.05). The 119S allele frequencies of *An*. *kleini* (0.66) and *An*. *sinensis* (0.67) collected in/near the DMZ were similar (Mann-Whitney U-tests, p > 0.05), but much higher than *An*. *belenrae* (0.19) and *An*. *pullus* (0.03) (Mann-Whitney U-tests, p < 0.01). The 119S resistant allele was not observed in *An*. *pullus* collected at Humphreys USAG. Three heterozygous-resistant and two homozygous-resistant alleles were identified in the *An*. *kleini*-*An*. *sinensis* hybrids collected in/near the DMZ. The HWE test identified a significant departure (heterozygote excess (p <0.01)) for the two populations of *An*. *sinensis* collected in/near the DMZ and Humphreys USAG (**Table 2**). Furthermore, the *An*. *kleini* population in/near the DMZ exhibited deviations from HWE, specifically showing heterozygote excess (p < 0.01). A significant proportion of 119S homozygotes was observed in both *An*. *sinensis* (29.4~35.2%) *and An*. *kleini* (34.0%), which aligns with previous findings in China [39]. In all eight species, only the GGC codon was identified in individuals with the 119G genotype.

**Table 2:** Number (%) of the *ace-1* genotypes and homozygous susceptible, heterozygous resistant, and homozygous resistant allele frequencies for each of the *Anopheles* species collected in/near the DMZ (6 collection sites), Yongsan USAG, and Humphreys USAG.

Collection sites	Species	N	*ace-1* genotypes** No. (%)			HWE	Allele frequency	
			119G/119G	119G/119S	119S/119S	Probability test (p-value)	119G	119S
In/near DMZ	*An*. *sinensis*	71	1 (1.4)	45 (63.4)	25 (35.2)	<0.01	0.33	0.67
	*An*. *kleini*	125	2 (1.6)	80 (64.0)	43 (34.0)	<0.01	0.34	0.66
	Hybrid*	5	0	3 (60.0)	2 (40.0)	-	0.30	0.70
	*An*. *belenrae*	48	31 (64.6)	16 (33.3)	1 (2.1)	>0.99	0.81	0.19
	*An*. *pullus*	54	51 (94.4)	3 (5.6)	0	>0.99	0.97	0.03
	*An*. *lesteri*	3	3 (100)	0	0	-	1	0
	*An*. *sineroides*	61	61 (100)	0	0	-	1	0
	*An*. *koreicus*	26	26 (100)	0	0	-	1	0
	*An*. *lindesayi*	8	8 (100)	0	0	-	1	0
Yongsan USAG	*An*. *sinensis*	17	1 (5.8)	11 (64.7)	5 (29.4)	0.30	0.38	0.62
	*An*. *lindesayi*	1	1 (100)	0	0	-	>0.99	<0.01
Humphreys USAG	*An*. *sinensis*	34	1 (2.9)	27 (79.4)	6 (29.4)	<0.01	0.43	0.57
	*An*. *pullus*	22	22 (100)	0	0	-	1	0
	*An*. *lesteri*	14	14 (100)	0	0	-	1	0

* Hybrid refers to *An*. *sinensis*–*An*. *kleini* hybrid individuals.

** 119G/119G = Homozygous susceptible; 119G/119S = Heterozygous resistant; 119S/119S = Homozygous resistant.

The ITS2 region in individuals carrying the insecticide resistance mutation was subsequently sequenced, revealing no disparities compared to the sequences of each documented species (GenBank accession numbers: *An*. *belenrae*: PP581930—PP581946; *An*. *pullus*: PP581921—PP581923; *An*. *kleini*: PP716866—PP716870).

## Detection of *kdr* mutations in *vgsc* gene fragments

Screening for *vgsc* gene fragments resulted in the detection of nine *kdr* genotypes (**S2 Fig**). The 1014F and 1014C (resistant) *kdr* alleles were exclusively identified in two species, *An*. *sinensis* and *An*. *kleini* (**Table 3**). The allele frequencies of 1014L, 1014F, and 1014C observed in *An*. *sinensis* in/near the DMZ, were 0.39, 0.36, and 0.25, respectively. Similarly, the 1014L, 1014F, and 1014C allele frequencies in the *An*. *sinensis* collected at Yongsan USAG were 0.35, 0.47, and 0.18, respectively, while the 1014L, 1014F, and 1014C allele frequencies for *An*. *sinensis* at Humphreys USAG were 0.28, 0.53, and 0.19, respectively. Among the three populations of *An*. *sinensis* examined, the Humphreys USAG specimens exhibited the highest *kdr* mutation (1014F+1014C) allele frequency (0.72), whereas the population in/near the DMZ showed the lowest frequency (0.61). However, no significant statistical differences were observed in the frequencies of resistance mutation alleles among the three regions of *An*. *sinensis* (Mann-Whitney U-tests, p > 0.05). The 1014L, 1014F, and 1014C allele frequencies for *An*. *kleini* collected in/near the DMZ were 0.97, 0.02, and 0.01, respectively, indicating a high level of susceptibility. All six individuals were identified as heterozygous resistant (1014L/1014F:5, 1014L/1014C:1) with no homozygous resistant individuals detected. Among the five *An*. *kleini*-*An*. *sinensis* hybrids, one exhibited homozygous susceptibility (1014L/1014L), while the other four displayed heterozygous resistance (1014L/1014F:1, 1014L/1014C:3). Similar to the findings of Kang *et al*. [26], *An*. *sineroides* exhibited the TTA (1014L) susceptibility allele, in contrast to the other seven species of *Anopheles* mosquitoes that harbored the TTG (1014L) allele. In addition, the TTC (1014F) allele was only found in *An*. *sinensis*. The HWE test confirmed that all three *An*. *sinensis* populations and *An*. *kleini* found in/near the DMZ conformed to HWE **(Table 3**).

**Table 3:** Number (%) of the *kdr* genotypes and homozygous susceptible, heterozygous resistant, and homozygous resistant allele frequencies for each of the *Anopheles* species collected in/near the DMZ (6 collection sites), Yongsan USAG, and Humphreys USAG.

Collection Sites	Species	N	*kdr* genotypes** No. (%)						HWE	Allele frequency		
			1014L/1014L	1014F/1014F	1014F/1014L	1014F/1014C	1014C/1014C	1014C/1014L	Probability test (p-value)	1014L	1014F	1014C
In/near DMZ	*An*. *sinensis*	71	10 (14.1)	9 (12.7)	21 (29.6)	12 (16.9)	4 (5.6)	15 (21.1)	>0.99	0.39	0.36	0.25
	*An*. *kleini*	125	119 (95.2)	0	5 (4.0)	0	0	1 (0.8)	>0.99	0.97	0.02	0.01
	Hybrid*	5	1 (20.0)	0	1 (20.0)	0	0	3 (60.0)	-	0.60	0.10	0.30
	*An*. *belenrae*	48	48 (100)	0	0	0	0	0	-	1	0	0
	*An*. *pullus*	54	54 (100)	0	0	0	0	0	-	1	0	0
	*An*. *lesteri*	3	3 (100)	0	0	0	0	0	-	1	0	0
	*An*. *sineroides*	61	61 (100)	0	0	0	0	0	-	1	0	0
	*An*. *koreicus*	26	26 (100)	0	0	0	0	0	-	1	0	0
	*An*. *lindesayi*	8	8 (100)	0	0	0	0	0	-	1	0	0
Yongsan USAG	*An*. *sinensis*	17	2 (11.8)	4 (23.5)	6 (35.2)	2 (11.8)	1 (5.9)	2 (11.8)	0.76	0.35	0.47	0.18
	*An*. *lindesayi*	1	1 (100)	0	0	0	0	0	-	1	0	0
Humphreys USAG	*An*. *sinensis*	34	3 (8.8)	11 (32.4)	8 (23.5)	6 (17.7)	1 (2.9)	5 (14.7)	0.75	0.28	0.53	0.19
	*An*. *pullus*	22	22 (100)	0	0	0	0	0	-	1	0	0
	*An*. *lesteri*	14	14 (100)	0	0	0	0	0	-	1	0	0

* Hybrid refers to *An*. *sinensis*–*An*. *kleini* hybrid individuals.

** 1014L (susceptible) = allele bases TTG and TTA; 1014C (resistant) = allele bases TGT; 1014F (resistant) = allele bases TTT and TTC

Further sequencing of the ITS2 region of *An*. *kleini*, where the *kdr* mutation was first detected, did not reveal any differences from the previously known sequence (GenBank accession numbers: *An*. *kleini*: PP581924—PP581929).

### Monthly variation in the 119S mutation

Monthly seasonal variations in the prevalence of *ace-1* 119S resistant alleles were determined for each species (**Tables 4 and S2**). For *An*. *sinensis* examined from June-October, the frequency of the 119S allele remained relatively stable (Mann-Whitney U-tests, June vs. October, p > 0.05), 0.75 (June), 0.62 (July), 0.64 (August), 0.61 (September), and 0.65 (October), respectively. *Anopheles kleini* was collected beginning in April, and both individuals collected exhibited homozygous resistance. In contrast to *An*. *sinensis*, from June-October, a period marked by significant population growth, the 119S resistant allele frequencies decreased monthly from 0.73 (June), 0.68 (July), 0.67 (August), 0.58 (September), to 0.50 (October) (Mann-Whitney U-tests, June vs. October, p < 0.05). *Anopheles sinensis*-*An*. *kleini* hybrids were collected only in October; two had homozygous resistance, while three had heterozygous resistance. 119S resistant alleles for *An*. *belenrae*, when >10 individuals were collected, were 0.23 (April), 0.33 (August), and 0.10 (October). For *An*. *pullus*, only one each for the months of April, August, and September were identified with 119S heterozygous resistant alleles.

**Table 4:** Monthly number (%) of homozygous susceptible, heterozygous resistant, and homozygous resistant of 119S alleles for *Anopheles* species. Insufficient numbers of *An*. *lesteri*, *An*. *koreicus* and *An*. *lindesayi* were collected and assayed for analysis.

Species	Month	N	*ace-1* genotypes No. (%)**			Allele frequency	
			119G/119G	119G/119S	119S/119S	119G	119S
*An*. *sinensis*	April	0	0	0	0	-	-
	May	0	0	0	0	-	-
	June	4	0	2 (50.0)	2 (50.0)	0.25	0.75
	July	12	1 (8.3)	7 (58.4)	4 (33.3)	0.38	0.62
	August	36	1 (2.8)	24 (66.7)	11 (30.5)	0.36	0.64
	September	44	0	34 (77.3)	10 (22.7)	0.39	0.61
	October	26	1 (3.8)	16 (61.6)	9 (34.6)	0.35	0.65
*An*. *kleini*	April	2	0	0	2 (100)	0	1
	May	1	0	1 (100)	0	0.50	0.50
	June	15	0	8 (53.3)	7 (46.7)	0.27	0.73
	July	39	0	25 (64.1)	14 (35.9)	0.32	0.68
	August	46	2 (4.4)	26 (56.5)	18 (39.1)	0.33	0.67
	September	13	0	11 (84.6)	2 (15.4)	0.42	0.58
	October	9	0	9 (100)	0	0.50	0.50
Hybrid*	April	0	0	0	0	-	-
	May	0	0	0	0	-	-
	June	0	0	0	0	-	-
	July	0	0	0	0	-	-
	August	0	0	0	0	-	-
	September	0	0	0	0	-	-
	October	5	0	3 (60.0)	2 (40.0)	0.3	0.7
*An*. *belenrae*	April	11	6 (54.5)	5 (45.5)	0	0.77	0.23
	May	2	1 (50.0)	1 (50.0)	0	0.75	0.25
	June	1	1 (100)	0	0	1	0
	July	1	0	1 (100)	0	0.50	0.50
	August	12	5 (41.7)	6 (50.0)	1 (8.3)	0.67	0.33
	September	6	6 (100)	0	0	1	0
	October	15	12 (80.0)	3 (20.0)	0	0.90	0.10
*An*. *pullus*	April	6	5 (83.3)	1 (16.7)	0	0.92	0.08
	May	23	23 (100)	0	0	1	0
	June	4	4 (100)	0	0	1	0
	July	7	7 (100)	0	0	1	0
	August	16	15 (93.8)	1 (6.2)	0	0.97	0.03
	September	12	11 (91.7)	1 (8.3)	0	0.96	0.04
	October	8	8 (100)	0	0	1	0
*An*. *sineroides*	April	20	20 (100)	0	0	1	0
	May	0	0	0	0	-	-
	June	11	11 (100)	0	0	1	0
	July	14	14 (100)	0	0	1	0
	August	4	4 (100)	0	0	1	0
	September	10	10 (100)	0	0	1	0
	October	2	2 (100)	0	0	1	0

* Hybrid refers to *An*. *sinensis*–*An*. *kleini* hybrid individuals.

** 119G/119G = Homozygous susceptible; 119G/119S = Heterozygous resistant; 119S/119S = Homozygous resistant.

### Monthly variation in the *kdr* mutations in *vgsc* gene fragments

The *kdr* resistant gene frequency (1014F+1014C) for *An*. *sinensis* ranged from 0.50 to 0.67 from July-October (**Tables 5 and S3**). No significant statistical differences were observed in the frequencies of seasonal resistance mutations (Mann-Whitney U-tests, July vs. October, p > 0.05). When 1014F and 1014C were analyzed separately, the 1014F allele frequency for *An*. *sinensis* ranged from 0.33 to 0.48 from July-October (Mann-Whitney U-tests, July vs. October, p > 0.05), while the 1014C allele frequency ranged from 0.17 to 0.23 from July-October (Mann-Whitney U-tests, July vs. October, p > 0.05). The first *kdr* mutation for *An*. *kleini* was detected in only one individual in September (1014L/1014F) and heterozygous resistance in five of nine individuals in October (1014L/1014F:4; 1014L/1014C:1). From June-August, a comprehensive analysis was conducted on a cohort of 100 individuals, demonstrating the absence of any *kdr* mutations. Only one of five hybrid *An*. *sinensis*-*An*. *kleini* specimens collected in October displayed homozygous susceptibility, whereas other four exhibited heterozygous resistance. No *kdr* mutations were detected in the remaining six species that were analyzed monthly.

**Table 5:** Monthly number (%) of homozygous susceptible, heterozygous resistant, and homozygous resistant of *kdr* alleles for *Anopheles* species. Insufficient numbers of *An*. *lesteri*, *An*. *koreicus* and *An*. *lindesayi* were collected and assayed for analysis.

Species	Month	N	*kdr* genotypes No. (%)**						Allele frequency		
			1014L/1014L	1014F/1014F	1014F/1014L	1014F/1014C	1014C/1014C	1014C/1014L	1014L	1014F	1014C
*An*.	April	0	0	0	0	0	0	0	-	-	-
*sinensis*	May	0	0	0	0	0	0	0	-	-	-
	June	4	0	0	1 (25.0)	1 (25.0)	2 (50.0)	0	0.13	0.25	0.62
	July	12	3 (25.0)	1 (8.3)	4 (33.3)	2 (16.7)	0	2 (16.7)	0.5	0.33	0.17
	August	36	4 (11.1)	8 (22.2)	10 (27.8)	8 (22.2)	0	6 (16.7)	0.33	0.48	0.19
	September	44	4 (9.1)	11 (25.0)	12 (27.3)	6 (13.6)	2 (4.5)	9 (20.5)	0.33	0.45	0.22
	October	26	4 (15.4)	4 (15.4)	8 (30.8)	3 (11.5)	2 (7.7)	5 (19.2)	0.4	0.37	0.23
*An*.	April	2	2 (100)	0	0	0	0	0	1	0	0
*kleini*	May	1	1 (100)	0	0	0	0	0	1	0	0
	June	15	15 (100)	0	0	0	0	0	1	0	0
	July	39	39 (100)	0	0	0	0	0	1	0	0
	August	46	46 (100)	0	0	0	0	0	1	0	0
	September	13	12 (92.3)	0	1 (7.7)	0	0	0	0.96	0.04	0
	October	9	4 (44.4)	0	4 (44.4)	0	0	1 (11.2)	0.72	0.22	0.06
Hybrid*	April	0	0	0	0	0	0	0	-	-	-
	May	0	0	0	0	0	0	0	-	-	-
	June	0	0	0	0	0	0	0	-	-	-
	July	0	0	0	0	0	0	0	-	-	-
	August	0	0	0	0	0	0	0	-	-	-
	September	0	0	0	0	0	0	0	-	-	-
	October	5	1 (20.0)	0	1 (20.0)	0	0	3 (60.0)	0.6	0.1	0.3
*An*. *belenrae*	April	11	11 (100)	0	0	0	0	0	1	0	0
	May	2	2 (100)	0	0	0	0	0	1	0	0
	June	1	1 (100)	0	0	0	0	0	1	0	0
	July	1	1 (100)	0	0	0	0	0	1	0	0
	August	12	12 (100)	0	0	0	0	0	1	0	0
	September	6	6 (100)	0	0	0	0	0	1	0	0
	October	15	15 (100)	0	0	0	0	0	1	0	0
*An*.	April	6	6 (100)	0	0	0	0	0	1	0	0
*pullus*	May	23	23 (100)	0	0	0	0	0	1	0	0
	June	4	4 (100)	0	0	0	0	0	1	0	0
	July	7	7 (100)	0	0	0	0	0	1	0	0
	August	16	16 (100)	0	0	0	0	0	1	0	0
	September	12	12 (100)	0	0	0	0	0	1	0	0
	October	8	8 (100)	0	0	0	0	0	1	0	0
*An*. *sineroides*	April	20	20 (100)	0	0	0	0	0	1	0	0
	May	0	0	0	0	0	0	0	-	-	-
	June	11	11 (100)	0	0	0	0	0	1	0	0
	July	14	14 (100)	0	0	0	0	0	1	0	0
	August	4	4 (100)	0	0	0	0	0	1	0	0
	September	10	10 (100)	0	0	0	0	0	1	0	0
	October	2	2 (100)	0	0	0	0	0	1	0	0

* Hybrid refers to *An*. *sinensis*–*An*. *kleini* hybrid individuals.

** 1014L (susceptible) = allele bases TTG and TTA; 1014C (resistant) = allele bases TGT; 1014F (resistant) = allele bases TTT and TTC.

**Fig 3:**
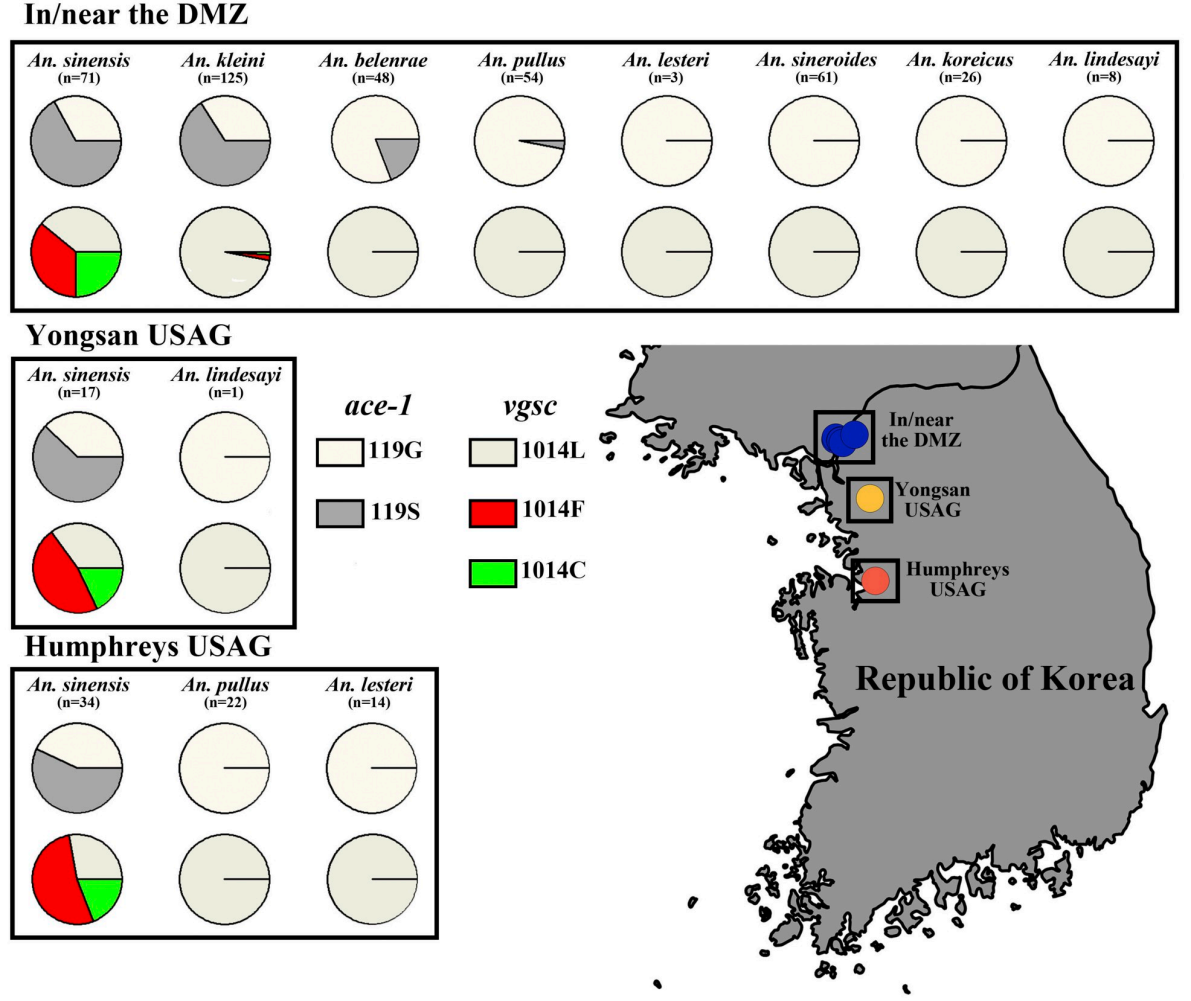
The proportion of *ace-1* and *kdr* allele frequencies in *Anopheles* species collected in/near the DMZ, Yongsan USAG, and Humphreys USAG. This map was created using QGIS version 3.26.3 (https://www.qgis.org/ko/site) and Natural Earth (https://www.naturalearthdata.com/downloads/10m-cultural-vectors; http://www.naturalearthdata.com/about/terms-of-use).

The ratio of both the 119S and *kdr* allele frequencies for *Anopheles* species collected in/near the DMZ, Yongsan USAG, and Humphreys USAG are shown in **Fig 3**.

## Discussion

A limited number of all eight species of *Anopheles* mosquitoes present in the ROK were examined for the presence of selected insecticide resistance mutations. While all eight species were collected in/near the DMZ, only two and three species were collected at Yongsan USAG and Humphreys USAG, respectively. These results are impacted by the predominance of each species over their range and environmental conditions, e.g. urbanization at Yongsan USAG and Humphreys USAG and environmental distributions, e.g. forested areas where *An*. *koreicus* and *An*. *lindesayi* predominant [45,46,47]. *Anopheles kleini* and *An*. *belenrae*, while collected throughout the ROK were more commonly collected in malaria high-risk areas in northern Gyeonggi provinces adjacent to the DMZ [26,48,49,50]. Significant variations in monthly occurrence densities among species of *Anopheles* have been recognized in the ROK [48,49,50]. Consequently, employing specimens collected during a specific season and location without precise species identification may result in biased results. Nevertheless, the limited sample size of *An*. *lesteri*, *An*. *koreicus*, and *An*. *lindesayi* specimens indicate the need for additional collections and analysis. *Anopheles lesteri* is noted for its high vector competence [8,9]. Additionally, *P*. *vivax* was initially identified in *An*. *lindesayi* by Lee *et al*. [50], emphasizing the necessity for ongoing surveillance to develop effective malaria control measures.

Pesticide resistance to organophosphates and carbamates has been associated with 119S pesticide resistant alleles in various mosquito species [51,52,53]. Thus, studies that identify pesticide resistant alleles in *Anopheles* spp. populations and their geographical and seasonal distributions are important for developing comprehensive malaria control measures, including the use of effective alternate pesticides. While the 119S resistant allele was previously observed in *An*. *sinensis* [24], it was detected for the first time in *An*. *kleini*, *An*. *belenrae*, and *An*. *pullus* in this study. In other studies, the 119S allele was observed in members of the Hyrcanus Group, but the results did not provide comparisons at the species level [28,29]. Although the highest frequency of the 119S allele in *An*. *sinensis* was observed in/near the DMZ, no statistically significant differences were detected when comparing the 119S allele frequencies in populations from other collection sites further south of the DMZ (Yongsan USAG, Humphreys USAG). The 119S allele frequency for *An*. *kleini* (0.66) collected in/near the DMZ was similar to *An*. *sinensis* (0.67). The 119S allele frequency of *An*. *belenrae* was much lower (0.19) and was only detected in *An*. *pullus* collected in/near the DMZ. High 119S allele frequencies have been observed in *An*. *sinensis* populations in China, a geographically neighboring country to the ROK [37,39,54,55]. The identification of 119S mutations in four of eight *Anopheles* species, particularly with high mutation rates in *An*. *sinensis* and *An*. *kleini*, implies that *Anopheles* mosquitoes in/near the DMZ likely experience significant selective insecticide pressures from the use of organophosphate and carbamate pesticides. This study observed departures from HWE among *An*. *sinensis* and *An*. *kleini* populations in/near the DMZ and *An*. *sinensis* populations at Humphreys USAG. This observation has also been documented in various populations of *An*. *sinensis* in China [39]. Qian. *et al*. [39] did not eliminate the possibility for the selection of heterozygotes; however, they proposed that heterologous duplication of the *ace-1* gene in *An*. *sinensis* might have occurred. Copy number variation in the *ace-1* gene has been previously documented in *An*. *gambiae*. Indeed, studies have demonstrated that the number of copies of *ace-1* also affects phenotype [40,56,57,58]. While copy number variation has not currently been explored in *An*. *sinensis*, our findings indicate the potential occurrence of copy number variations in *An*. *kleini*, similar to *An*. *sinensis*. The correlation between the presence or absence of copy number variations and insecticide resistance has significant implications for developing future effective vector control strategies [40,57,58]. Advancements in molecular biology techniques, coupled with reduced expenses, have facilitated the identification of copy number variations using various methodologies, including qPCR, droplet digital qPCR, fluorescent *in situ* hybridization, and next-generation sequencing [40]. Hence, it is important to investigate the copy number variations of *ace-1* mutations in *An*. *sinensis*, *An*. *kleini* (primary malaria vector in the ROK) and other *Anopheles* spp. as well as the molecular phenotyping of the *ace-1* gene (heterogeneous/homogeneous) to enhance vector control efficiency [59].

Currently, studies in the ROK have primarily concentrated on detecting *kdr* mutations in *An*. *sinensis* or broadly in combined members of the Hyrcanus Group [25,26,27,28,29,30]. Herein, we analyzed the species/individual level to address the constraints of prior experiments and identified the first *kdr* mutation in *An*. *kleini* in the ROK. Consistent with previous studies, we have reaffirmed the high prevalence of the 1014F and 1014C mutations in *An*. *sinensis*, with some regional variation. For *An*. *sinensis*, 1014F and 1014C were identified in all three regions; however, no regional trends were observed, which is consistent with previous studies [26]. In addition, the 1014S allele has been identified in *An*. *sinensis* populations in China [39]; however, it has not yet been detected in populations in the ROK. China is geographically close to the ROK, which raises the possibility for the introduction of the 1014S allele. Ongoing surveillance for the introduction of resistance mutations and their association with pyrethroid insecticides is necessary to adjust methodologies for an effective vector control program in the future. A study identified the heterozygous 1014F allele in 1 of 3 *An*. *belenrae* collected near the DMZ in 2022, while our study did not detect any *kdr* mutations in 48 *An*. *belenrae* collected during 2021 [30]. Based on these findings, *kdr* mutations appear at a low frequency in *An*. *belenrae* populations in the ROK. Similarly, molecular surveillance for *kdr* mutations in 30 *An*. *kleini* specimens collected from Yeoncheon, Gimpo, and Pyeongchang, an area adjacent to the DMZ, did not find any *kdr* resistant alleles [26]. The findings from this study indicate that none of the specimens examined carried the *kdr* mutation. Our analysis showed that all 103 specimens of *An*. *kleini* collected from April–August was negative, while 6/22 individuals collected during September-October carried the *kdr* pesticide resistant allele. The reason for the delayed detection of the *kdr* mutant allele in October is currently difficult to ascertain. However, this delay may be attributed to significant selection pressure against pyrethroid insecticides prior to October when the population experiences substantial growth (June–September). Furthermore, the high frequency of the 119S allele in *An*. *kleini*, coupled with the observation that the *kdr* mutation was exclusively detected in *An*. *sinensis* and a limited number of *An*. *kleini* specimens, in contrast to the 119S mutation present in four different species, implies a potentially higher exposure of selected *Anopheles* species to organophosphate insecticides. Previous studies showed that the highest number of larvae belonging to members of the Hyrcanus Group were reported to be found in rice paddies [47]. Organophosphate, carbamate, and pyrethroid insecticides are commonly employed in agricultural practices for pest management and may serve as a significant selective pressure for the *ace-1* mutant allele [60]. At present, assessing primary larval habitats and geographical and seasonal distributions is challenging; thus, further inquiry into the underlying causes of pesticide resistant alleles remains necessary.

Lee *et al*. [28] identified a pattern of seasonal changes for members of the Hyrcanus Group by detecting monthly 119S and *kdr* mutations. They observed that mutation frequencies were not observed in *Anopheles* mosquitoes belonging to the Hyrcanus group from May–June, yet gradually increased in the subsequent summer months before decreasing again in the fall. However, our results are inconsistent with these findings [28]. Both *An*. *sinensis* and *An*. *kleini* exhibited a consistent or elevated mutation frequency (>0.50) irrespective of the month collected. The monthly distribution of *kdr* mutations (1014F+1014C) in *An*. *sinensis*, from July (0.50) to October (0.60), showed a peak in August (0.67) to September (0.67) and a slight decrease in October (0.60). However, the mutation frequency remained above a constant level (>0.50) for each of the months, and no statistically significant differences were observed between seasons. The disparate findings between this study and earlier studies are likely attributable to variations in species monthly geographical and seasonal distributions. Lee *et al*. [28] used pooled individuals for their experiments rather than species-level comparisons. Making comparisons without precise species identification likely introduces bias in interpreting specific mutant alleles as *Anopheles* mosquitoes in the ROK demonstrate interspecific variability in monthly densities, as reported by previous studies [48,49,50]. Our findings also indicate potential variations in the seasonal prevalence of resistance 119S mutation across different species. In contrast to *An*. *sinensis*, which maintained a consistent frequency of 0.62 in July and 0.65 in October, *An*. *kleini* exhibited a frequency of 0.73 among individuals collected in June. However, it subsequently experienced a gradual decrease, resulting in a 119S mutation frequency of 0.50 in October. Investigations and molecular phenotyping of the copy number variation of the *ace-1* gene in *An*. *sinensis* and *An*. *kleini* are necessary before making definitive conclusions regarding hypotheses behind interspecies variations [40,57,58,59]. The outcomes of this study unequivocally advocate for the necessity of comparative analysis for each species collected over the entire mosquito seasons, as opposed to a single month when examining the retention of insecticide resistance mutations.

In this experiment, *An*. *sinensis*–*An*. *kleini* hybrid individuals were only found in October, and five out of six *An*. *kleini* individuals carrying the *kdr* mutation were also found in October. Joshi *et al*. [43] also identified hybrids among samples collected in September. While the possibility of chance cannot be ruled out, it seems likely that there are selection pressures driving hybridization from June through September. For *An*. *sinensis* and *An*. *kleini*, backcrossing is possible [44]. Gene flow and adaptive introgression are important factors for effective vector control [61,62,63], and these possibilities should be explored in the future.

Studies that identify seasonal and geographical distributions, in addition to the prevalence of pesticide resistant alleles that impact on effective malaria vector control measures should be considered in the future. Bioassays were not conducted in this study to confirm the association between target site mutations and insecticide resistance. As this study has identified resistance mutations in previously unknown species, it is anticipated that bioassays will be necessary for future validation. Vector control measures are important criteria for the WHO objectives to eliminate vivax malaria from the ROK by 2028.

## Conclusion

In this study, we investigated eight species of *Anopheles* mosquitoes in the ROK to determine the presence or absence of the 119S mutation in *ace-1* and the *kdr* mutation in *vgsc* gene fragments. In contrast to the 119S mutation, which was detected in four of the eight species, the *kdr* mutation was exclusively observed in *An*. *sinensis* and *An*. *kleini*. Differences in monthly mutation allele frequencies among species were also observed. This indicates that advanced control methods, such as rotating and mixing insecticides, are necessary for effective vector management. It also emphasizes the necessity of developing new control strategies, such as bio-control, that can complement the use of insecticides. Furthermore, the duplication of *ace-1* and the potential for gene exchange or adaptive introgression between *An*. *sinensis* and *An*. *kleini* requires immediate attention in the management of malaria cases. The mosquito collection sites were located within prominent malaria high-risk military zones in the ROK. It is noteworthy that historically, diseases transmitted by mosquitoes have exerted a substantial influence on military capabilities [64]. The results of this experiment are anticipated to make a significant contribution to the advancement of forthcoming malaria control strategies. Since insecticide resistance poses a substantial risk to public health, ongoing surveillance and research efforts are imperative.

## Supporting information

S1 Fig Example of a chromatogram for the *ace-1* gene. Homozygous resistance, AGC/AGC (119S/119S) (**A)**. Heterozygous resistance, GGC/AGC (119G/119S). (**B**). Homozygous susceptibility, GGC/GGC (119G/119G) (**C**).
(TIF)

S2 Fig Example of a chromatogram for the *vgsc* gene. Homozygous susceptibility, TTG/TTG (1014L/1014L) (**A**). Homozygous susceptibility, TTA/TTA (1014L/1014L) (**B**). Homozygous resistance, TTT/TTT (11014F/11014F) (**C)**. Homozygous resistance, TGT/TGT (11014C/11014C) (**D)**. Heterozygous resistance, TTT/TTC (1014F/1014F) (**E**). Heterozygous resistance, TTT/TTG (1014F/1014L) (**F**). Heterozygous resistance, TTT/TGT (1014F/1014C) (**G**). Heterozygous resistance, TGT/TTG (1014C/1014L) (**H**). Heterozygous resistance, TTC/TTG (1014F/1014L) (**I**).
(TIF)

S1 Table Information about *Anopheles* collection sites. (DOCX)

S2 Table Monthly variation in the frequency of the G119S mutation across different species. (DOCX)

S3 Table Monthly variation in the frequency of the *kdr* mutation across different species. (DOCX)
