# Supplementary figures and images for: Insecticide resistance mutations of Anopheles species in the Republic of Korea
Source: PLoS Negl Trop Dis. 2025 Jan 7;19(1):e0012748. doi: 10.1371/journal.pntd.0012748 (PMC11706468; doi:10.1371/journal.pntd.0012748)

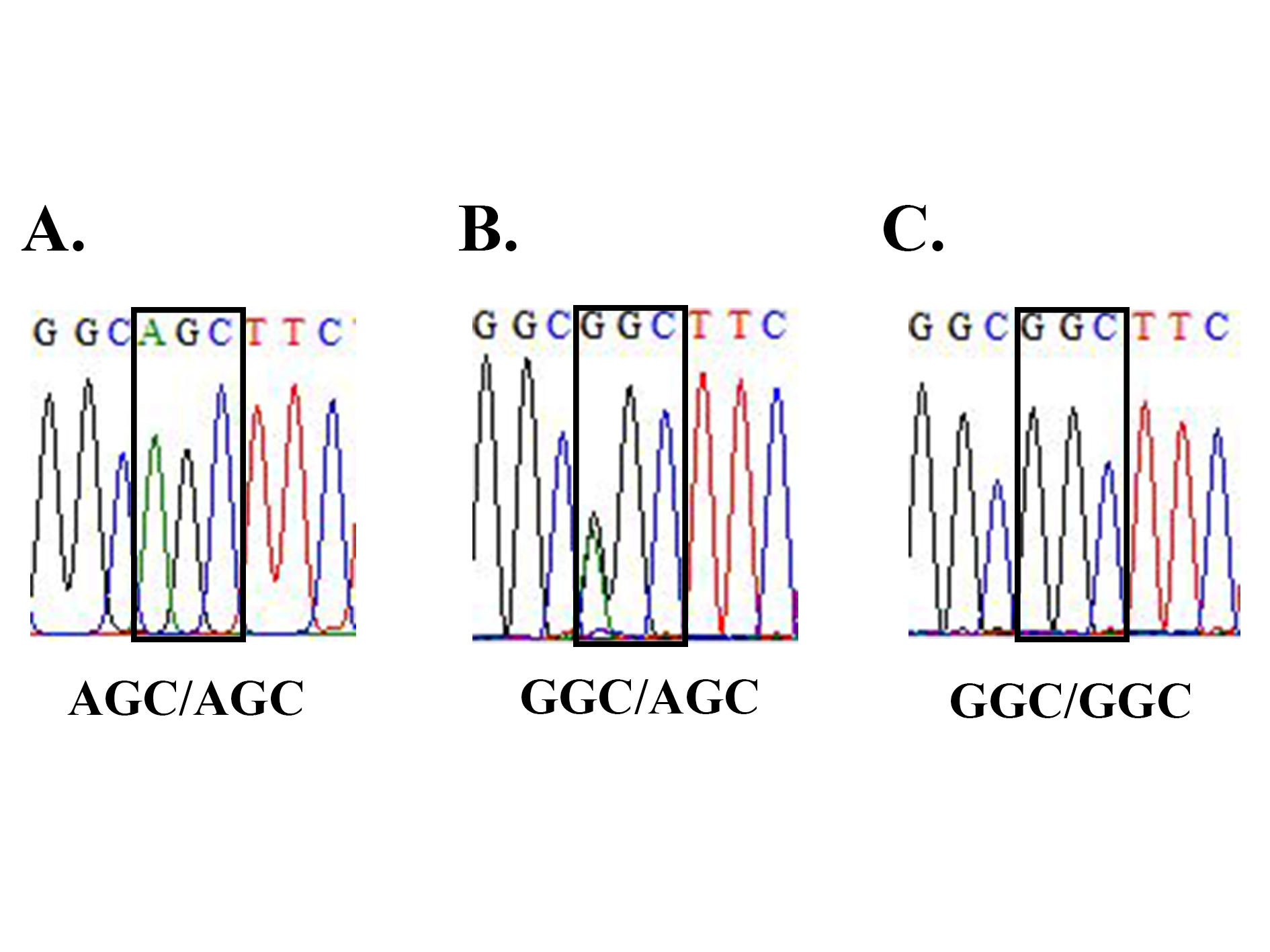

Supplement: S1 Fig — Homozygous resistance, AGC/AGC (119S/119S) (A). Heterozygous resistance, GGC/AGC (119G/119S). (B). Homozygous susceptibility, GGC/GGC (119G/119G) (C). (TIF) [file pntd.0012748.s001.tif]

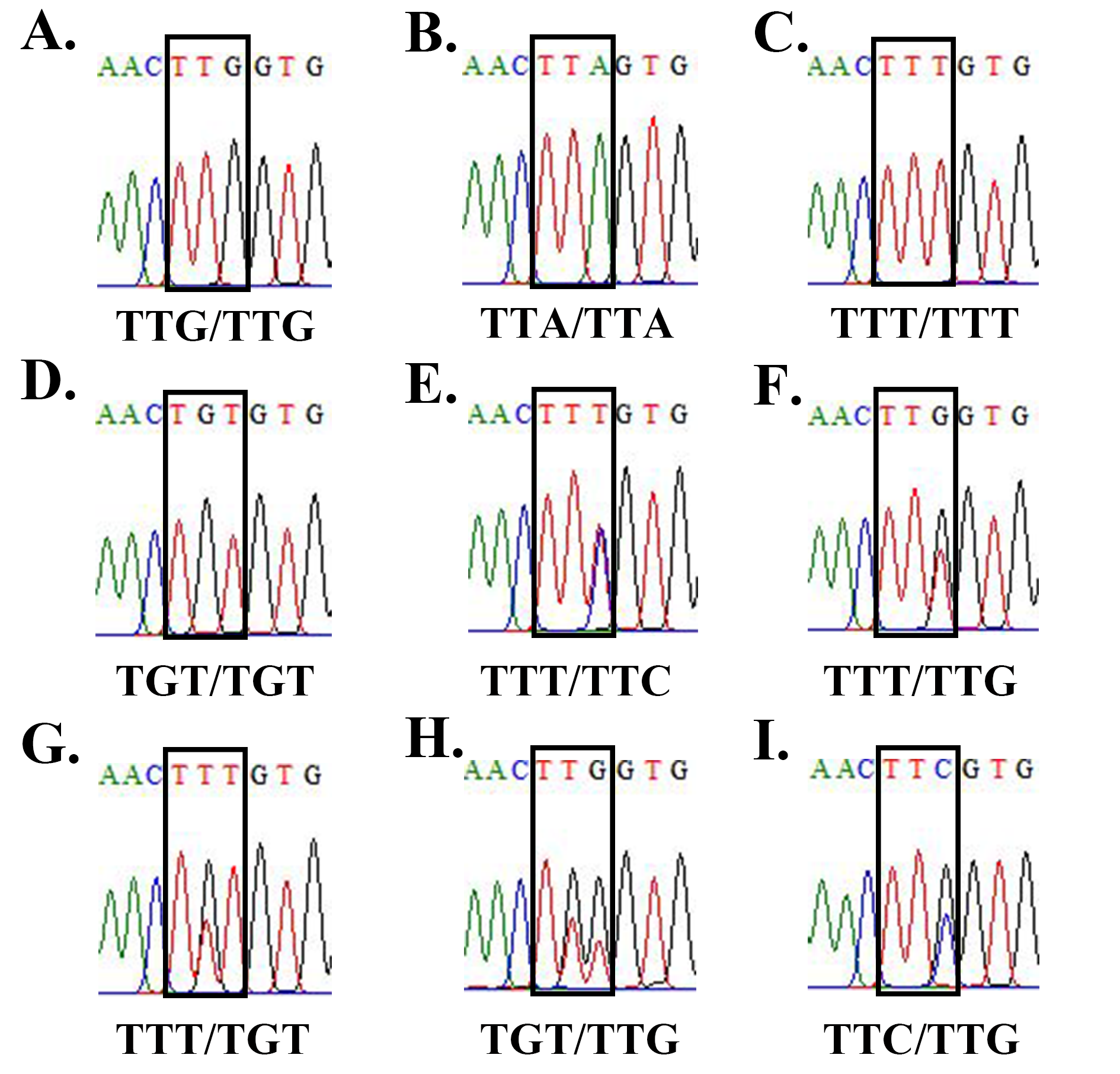

Supplement: S2 Fig — Homozygous susceptibility, TTG/TTG (1014L/1014L) (A). Homozygous susceptibility, TTA/TTA (1014L/1014L) (B). Homozygous resistance, TTT/TTT (11014F/11014F) (C). Homozygous resistance, TGT/TGT (11014C/11014C) (D). Heterozygous resistance, TTT/TTC (1014F/1014F) (E). Heterozygous resistance, TTT/TTG (1014F/1014L) (F). Heterozygous resistance, TTT/TGT (1014F/1014C) (G). Heterozygous resistance, TGT/TTG (1014C/1014L) (H). Heterozygous resistance, TTC/TTG (1014F/1014L) (I). (TIF) [file pntd.0012748.s002.tif]
