# Supplementary material for: Insecticide resistance mutations of Anopheles species in the Republic of Korea
Source: PLoS Negl Trop Dis. 2025 Jan 7;19(1):e0012748. doi: 10.1371/journal.pntd.0012748 (PMC11706468; doi:10.1371/journal.pntd.0012748)
Supplement: S1 Table — (DOCX) [file pntd.0012748.s003.docx]

**S1 Table.** Information about *Anopheles* collection sites.

| Collection site | | Coordinates | Environmental feature |
| --- | --- | --- | --- |
| In/near the DMZ | Neutral Nations Supervisory Commission camp | 37°57ʹ17.19″ N 126°40ʹ47.91″ E | Two Traps were set among deciduous trees adjacent to NNSC housing and an open area adjacent to a low-lying area, both <10 m from the DMZ bordering North and South Korea. |
|  | Daeseong-dong | 37°56ʹ28.31″ N 126°40ʹ37.38″ | One trap was set 10 m from the village and adjacent to rice paddies extending to the DMZ. |
|  | South gate entrance to the DMZ | 37°56ʹ03.53″ N 126°43ʹ15.46″ E | One trap was set adjacent to a mixed forest and 10 m from the guard station and low-lying grassy stream. |
|  | Camp Bonifas | 37°55ʹ55.25″ N 126°43ʹ21.73″ E | One trap was set among a grove of pine trees adjacent to Officer barracks. |
|  | Warrior Base training area | 37°55ʹ03.96″ N 126°44ʹ29.74″ E | Two traps were set on the periphery of office and barrack building and adjacent to extensive rice paddies and associated drainage ditches. |
|  | Dagmar North training area | 37°58ʹ29.85″ N 126°50ʹ40.88″ E | Two traps were set among deciduous trees and approximately 100 m from the Imjin River and low-lying grassy areas that flooded during rains. |
| Non-DMZ | Yongsan US  Army Garrison | 37°31ʹ56.2″ N 126°58ʹ53.4″ E | Two traps were set adjacent to a forested area bordering military/housing buildings and near drainage ditches that often-held water during raining periods. |
|  | Pyeongtaek US Army Garrison | 36°57ʹ19.9″ N 127°01ʹ41.4″ E | A total of 12 traps were set along the perimeter of Humphreys USAG among military/housing buildings and a golf course with small/large drainage systems. Traps were also set adjacent to the Anseong River with extensive grassy margins. |
