# Supplementary material for: Insecticide resistance mutations of Anopheles species in the Republic of Korea
Source: PLoS Negl Trop Dis. 2025 Jan 7;19(1):e0012748. doi: 10.1371/journal.pntd.0012748 (PMC11706468; doi:10.1371/journal.pntd.0012748)
Supplement: S2 Table — (DOCX) [file pntd.0012748.s004.docx]

**S2 Table.** Monthly variation in the frequency of the G119S mutation across different species.

| Species | Month | N | *ace-1* genotypes | | | Allele frequency | |
| --- | --- | --- | --- | --- | --- | --- | --- |
|  |  |  | GGC/GGC | GGC/AGC | AGC/AGC | G119 | G119S |
| *An. sinensis* | April | 0 | 0 | 0 | 0 | - | - |
|  | May | 0 | 0 | 0 | 0 | - | - |
|  | June | 4 | 0 | 2 | 2 | 0.25 | 0.75 |
|  | July | 12 | 1 | 7 | 4 | 0.38 | 0.62 |
|  | August | 36 | 1 | 24 | 11 | 0.36 | 0.64 |
|  | September | 44 | 0 | 34 | 10 | 0.39 | 0.61 |
|  | October | 26 | 1 | 16 | 9 | 0.35 | 0.65 |
| *An. kleini* | April | 2 | 0 | 0 | 2 | 0 | 1.00 |
|  | May | 1 | 0 | 1 | 0 | 0.50 | 0.50 |
|  | June | 15 | 0 | 8 | 7 | 0.27 | 0.73 |
|  | July | 39 | 0 | 25 | 14 | 0.32 | 0.68 |
|  | August | 46 | 2 | 26 | 18 | 0.33 | 0.67 |
|  | September | 13 | 0 | 11 | 2 | 0.42 | 0.58 |
|  | October | 9 | 0 | 9 | 0 | 0.50 | 0.50 |
| Hybrid* | April | 0 | 0 | 0 | 0 | - | - |
|  | May | 0 | 0 | 0 | 0 | - | - |
|  | June | 0 | 0 | 0 | 0 | - | - |
|  | July | 0 | 0 | 0 | 0 | - | - |
|  | August | 0 | 0 | 0 | 0 | - | - |
|  | September | 0 | 0 | 0 | 0 | - | - |
|  | October | 5 | 0 | 3 | 2 | 0.30 | 0.70 |
| *An. belenrae* | April | 11 | 6 | 5 | 0 | 0.77 | 0.23 |
|  | May | 2 | 1 | 1 | 0 | 0.75 | 0.25 |
|  | June | 1 | 1 | 0 | 0 | 1.00 | 0 |
|  | July | 1 | 0 | 1 | 0 | 0.50 | 0.50 |
|  | August | 12 | 5 | 6 | 1 | 0.67 | 0.33 |
|  | September | 6 | 6 | 0 | 0 | 1.00 | 0 |
|  | October | 15 | 12 | 3 | 0 | 0.90 | 0.10 |
| *An. pullus* | April | 6 | 5 | 1 | 0 | 0.92 | 0.08 |
|  | May | 23 | 23 | 0 | 0 | 1.00 | 0 |
|  | June | 4 | 4 | 0 | 0 | 1.00 | 0 |
|  | July | 7 | 7 | 0 | 0 | 1.00 | 0 |
|  | August | 16 | 15 | 1 | 0 | 0.97 | 0.03 |
|  | September | 12 | 11 | 1 | 0 | 0.96 | 0.04 |
|  | October | 8 | 8 | 0 | 0 | 1.00 | 0 |
| *An. lesteri* | April | 0 | 0 | 0 | 0 | - | - |
|  | May | 10 | 10 | 0 | 0 | 1.00 | 0 |
|  | June | 3 | 3 | 0 | 0 | 1.00 | 0 |
|  | July | 0 | 0 | 0 | 0 | - | - |
|  | August | 0 | 0 | 0 | 0 | - | - |
|  | September | 0 | 0 | 0 | 0 | - | - |
|  | October | 4 | 4 | 0 | 0 | 1.00 | 0 |
| *An. sineroides* | April | 20 | 20 | 0 | 0 | 1.00 | 0 |
|  | May | 0 | 0 | 0 | 0 | - | - |
|  | June | 11 | 11 | 0 | 0 | 1.00 | 0 |
|  | July | 14 | 14 | 0 | 0 | 1.00 | 0 |
|  | August | 4 | 4 | 0 | 0 | 1.00 | 0 |
|  | September | 10 | 10 | 0 | 0 | 1.00 | 0 |
|  | October | 2 | 2 | 0 | 0 | 1.00 | 0 |
| *An. koreicus* | April | 5 | 5 | 0 | 0 | 1.00 | 0 |
|  | May | 2 | 2 | 0 | 0 | 1.00 | 0 |
|  | June | 0 | 0 | 0 | 0 | - | - |
|  | July | 10 | 10 | 0 | 0 | 1.00 | 0 |
|  | August | 0 | 0 | 0 | 0 | - | - |
|  | September | 5 | 5 | 0 | 0 | 1.00 | 0 |
|  | October | 4 | 4 | 0 | 0 | 1.00 | 0 |
| *An. lindesayi* | April | 0 | 0 | 0 | 0 | - | - |
|  | May | 0 | 0 | 0 | 0 | - | - |
|  | June | 1 | 1 | 0 | 0 | 1.00 | 0 |
|  | July | 2 | 2 | 0 | 0 | 1.00 | 0 |
|  | August | 0 | 0 | 0 | 0 | - | - |
|  | September | 0 | 0 | 0 | 0 | - | - |
|  | October | 6 | 6 | 0 | 0 | 1.00 | 0 |

* Hybrid refers to *An. sinensis*/*An. kleini* hybrid individuals.

** GGC/GGC= Homozygous susceptible; GGC/AGC = Heterozygous resistant; AGC/AGC = Homozygous resistant.

*** 119G (susceptible) = allele bases GGC; 119S (resistant) = allele bases AGC
