# Supplementary material for: Insecticide resistance mutations of Anopheles species in the Republic of Korea
Source: PLoS Negl Trop Dis. 2025 Jan 7;19(1):e0012748. doi: 10.1371/journal.pntd.0012748 (PMC11706468; doi:10.1371/journal.pntd.0012748)
Supplement: S3 Table — (DOCX) [file pntd.0012748.s005.docx]

**S3 Table.** Monthly variation in the frequency of the *kdr* mutation across different species.

| Species | Month | N | *vgsc* genotypes | | | | | | | | | Allele frequency | | |
| --- | --- | --- | --- | --- | --- | --- | --- | --- | --- | --- | --- | --- | --- | --- |
|  |  |  | TTG/TTG | TTA/TTA | TTT/TTT | TTT/TTC | TTT/TTG | TTT/TGT | TGT/TGT | TGT/TTG | TTC/TTG | 1014L | 1014F | 1014C |
| *An. sinensis* | April | 0 | 0 | 0 | 0 | 0 | 0 | 0 | 0 | 0 | 0 | - | - | - |
|  | May | 0 | 0 | 0 | 0 | 0 | 0 | 0 | 0 | 0 | 0 | - | - | - |
|  | June | 4 | 0 | 0 | 0 | 0 | 1 | 1 | 2 | 0 | 0 | 0.13 | 0.25 | 0.62 |
|  | July | 12 | 3 | 0 | 1 | 0 | 4 | 2 | 0 | 2 | 0 | 0.50 | 0.33 | 0.17 |
|  | August | 36 | 4 | 0 | 7 | 1 | 9 | 8 | 0 | 6 | 1 | 0.33 | 0.48 | 0.19 |
|  | September | 44 | 4 | 0 | 9 | 2 | 12 | 6 | 2 | 9 | 0 | 0.33 | 0.45 | 0.22 |
|  | October | 26 | 4 | 0 | 4 | 0 | 7 | 3 | 2 | 5 | 1 | 0.40 | 0.37 | 0.23 |
| *An. kleini* | April | 2 | 2 | 0 | 0 | 0 | 0 | 0 | 0 | 0 | 0 | 1.00 | 0 | 0 |
|  | May | 1 | 1 | 0 | 0 | 0 | 0 | 0 | 0 | 0 | 0 | 1.00 | 0 | 0 |
|  | June | 15 | 15 | 0 | 0 | 0 | 0 | 0 | 0 | 0 | 0 | 1.00 | 0 | 0 |
|  | July | 39 | 39 | 0 | 0 | 0 | 0 | 0 | 0 | 0 | 0 | 1.00 | 0 | 0 |
|  | August | 46 | 46 | 0 | 0 | 0 | 0 | 0 | 0 | 0 | 0 | 1.00 | 0 | 0 |
|  | September | 13 | 12 | 0 | 0 | 0 | 1 | 0 | 0 | 0 | 0 | 0.96 | 0.04 | 0 |
|  | October | 9 | 4 | 0 | 0 | 0 | 4 | 0 | 0 | 1 | 0 | 0.72 | 0.22 | 0.06 |
| Hybrid* | April | 0 | 0 | 0 | 0 | 0 | 0 | 0 | 0 | 0 | 0 | - | - | - |
|  | May | 0 | 0 | 0 | 0 | 0 | 0 | 0 | 0 | 0 | 0 | - | - | - |
|  | June | 0 | 0 | 0 | 0 | 0 | 0 | 0 | 0 | 0 | 0 | - | - | - |
|  | July | 0 | 0 | 0 | 0 | 0 | 0 | 0 | 0 | 0 | 0 | - | - | - |
|  | August | 0 | 0 | 0 | 0 | 0 | 0 | 0 | 0 | 0 | 0 | - | - | - |
|  | September | 0 | 0 | 0 | 0 | 0 | 0 | 0 | 0 | 0 | 0 | - | - | - |
|  | October | 5 | 1 | 0 | 0 | 0 | 1 | 0 | 0 | 3 | 0 | 0.60 | 0.10 | 0.30 |
| *An. belenrae* | April | 11 | 11 | 0 | 0 | 0 | 0 | 0 | 0 | 0 | 0 | 1.00 | 0 | 0 |
|  | May | 2 | 2 | 0 | 0 | 0 | 0 | 0 | 0 | 0 | 0 | 1.00 | 0 | 0 |
|  | June | 1 | 1 | 0 | 0 | 0 | 0 | 0 | 0 | 0 | 0 | 1.00 | 0 | 0 |
|  | July | 1 | 1 | 0 | 0 | 0 | 0 | 0 | 0 | 0 | 0 | 1.00 | 0 | 0 |
|  | August | 12 | 12 | 0 | 0 | 0 | 0 | 0 | 0 | 0 | 0 | 1.00 | 0 | 0 |
|  | September | 6 | 6 | 0 | 0 | 0 | 0 | 0 | 0 | 0 | 0 | 1.00 | 0 | 0 |
|  | October | 15 | 15 | 0 | 0 | 0 | 0 | 0 | 0 | 0 | 0 | 1.00 | 0 | 0 |
| *An. pullus* | April | 6 | 6 | 0 | 0 | 0 | 0 | 0 | 0 | 0 | 0 | 1.00 | 0 | 0 |
|  | May | 23 | 23 | 0 | 0 | 0 | 0 | 0 | 0 | 0 | 0 | 1.00 | 0 | 0 |
|  | June | 4 | 4 | 0 | 0 | 0 | 0 | 0 | 0 | 0 | 0 | 1.00 | 0 | 0 |
|  | July | 7 | 7 | 0 | 0 | 0 | 0 | 0 | 0 | 0 | 0 | 1.00 | 0 | 0 |
|  | August | 16 | 16 | 0 | 0 | 0 | 0 | 0 | 0 | 0 | 0 | 1.00 | 0 | 0 |
|  | September | 12 | 12 | 0 | 0 | 0 | 0 | 0 | 0 | 0 | 0 | 1.00 | 0 | 0 |
|  | October | 8 | 8 | 0 | 0 | 0 | 0 | 0 | 0 | 0 | 0 | 1.00 | 0 | 0 |
| *An. lesteri* | April | 0 | 0 | 0 | 0 | 0 | 0 | 0 | 0 | 0 | 0 | - | - | - |
|  | May | 10 | 10 | 0 | 0 | 0 | 0 | 0 | 0 | 0 | 0 | 1.00 | 0 | 0 |
|  | June | 3 | 3 | 0 | 0 | 0 | 0 | 0 | 0 | 0 | 0 | 1.00 | 0 | 0 |
|  | July | 0 | 0 | 0 | 0 | 0 | 0 | 0 | 0 | 0 | 0 | - | - | - |
|  | August | 0 | 0 | 0 | 0 | 0 | 0 | 0 | 0 | 0 | 0 | - | - | - |
|  | September | 0 | 0 | 0 | 0 | 0 | 0 | 0 | 0 | 0 | 0 | - | - | - |
|  | October | 4 | 4 | 0 | 0 | 0 | 0 | 0 | 0 | 0 | 0 | 1.00 | 0 | 0 |
| *An. sineroides* | April | 20 | 0 | 20 | 0 | 0 | 0 | 0 | 0 | 0 | 0 | 1.00 | 0 | 0 |
|  | May | 0 | 0 | 0 | 0 | 0 | 0 | 0 | 0 | 0 | 0 | - | - | - |
|  | June | 11 | 0 | 11 | 0 | 0 | 0 | 0 | 0 | 0 | 0 | 1.00 | 0 | 0 |
|  | July | 14 | 0 | 14 | 0 | 0 | 0 | 0 | 0 | 0 | 0 | 1.00 | 0 | 0 |
|  | August | 4 | 0 | 4 | 0 | 0 | 0 | 0 | 0 | 0 | 0 | 1.00 | 0 | 0 |
|  | September | 10 | 0 | 10 | 0 | 0 | 0 | 0 | 0 | 0 | 0 | 1.00 | 0 | 0 |
|  | October | 2 | 0 | 2 | 0 | 0 | 0 | 0 | 0 | 0 | 0 | 1.00 | 0 | 0 |
| *An. koreicus* | April | 5 | 5 | 0 | 0 | 0 | 0 | 0 | 0 | 0 | 0 | 1.00 | 0 | 0 |
|  | May | 2 | 2 | 0 | 0 | 0 | 0 | 0 | 0 | 0 | 0 | 1.00 | 0 | 0 |
|  | June | 0 | 0 | 0 | 0 | 0 | 0 | 0 | 0 | 0 | 0 | - | - | - |
|  | July | 10 | 10 | 0 | 0 | 0 | 0 | 0 | 0 | 0 | 0 | 1.00 | 0 | 0 |
|  | August | 0 | 0 | 0 | 0 | 0 | 0 | 0 | 0 | 0 | 0 | - | - | - |
|  | September | 5 | 5 | 0 | 0 | 0 | 0 | 0 | 0 | 0 | 0 | 1.00 | 0 | 0 |
|  | October | 4 | 4 | 0 | 0 | 0 | 0 | 0 | 0 | 0 | 0 | 1.00 | 0 | 0 |
| *An. lindesayi* | April | 0 | 0 | 0 | 0 | 0 | 0 | 0 | 0 | 0 | 0 | - | - | - |
|  | May | 0 | 0 | 0 | 0 | 0 | 0 | 0 | 0 | 0 | 0 | - | - | - |
|  | June | 1 | 1 | 0 | 0 | 0 | 0 | 0 | 0 | 0 | 0 | 1.00 | 0 | 0 |
|  | July | 2 | 2 | 0 | 0 | 0 | 0 | 0 | 0 | 0 | 0 | 1.00 | 0 | 0 |
|  | August | 0 | 0 | 0 | 0 | 0 | 0 | 0 | 0 | 0 | 0 | - | - | - |
|  | September | 0 | 0 | 0 | 0 | 0 | 0 | 0 | 0 | 0 | 0 | - | - | - |
|  | October | 6 | 6 | 0 | 0 | 0 | 0 | 0 | 0 | 0 | 0 | 1.00 | 0 | 0 |

* Hybrid refers to *An. sinensis*–*An. kleini* hybrid individuals.

** TTG/TTG and TTA/TTA = Homozygous susceptible; TTT/TTC, TTT/TTG, TTT/TGT, TGT/TTG and TTC/TTG = Heterozygous resistant; TTT/TTT and TGT/TGT= Homozygous resistant.

*** 1014L (susceptible) = allele bases TTG and TTA; 1014C (resistant) = allele bases TGT; 1014F (resistant) = allele bases TTT and TTC
